# Testing the study protocol and interrater reliability of a new end-of-life wound assessment tool: a feasibility study

**DOI:** 10.1186/s12904-025-01853-9

**Published:** 2025-07-29

**Authors:** Sharon Latimer, Rachel M. Walker, Jayne Hewitt, Gillian Ray-Barruel, Joanie Shaw, Tracey Hunt, Brigid M. Gillespie

**Affiliations:** 1 School of Nursing and Midwifery, and NHMRC Centre of Research Excellence in Wiser Wounds Care, Griffith University, Southport, QLD Australia; 2 College of Healthcare Sciences Academy, James Cook University, Townsville, QLD Australia; 3 Townsville Institute of Health Research & Innovation, Townsville University Hospital, Townsville, QLD Australia; 4 School of Nursing and Midwifery, Griffith University, Southport, QLD Australia; 5 Queen Elizabeth II Jubilee Hospital, Brisbane, QLD Australia; 6 School of Nursing, Midwifery and Social Work, University of Queensland, Brisbane, QLD Australia; 7 Herston Infectious Diseases Institute, Metro North Health, Brisbane, QLD Australia; 8 School of Nursing and Midwifery, Griffith University, Nathan, QLD Australia; 9 Cancer, Blood Disorders, Specialist Palliative Care, Immunology & Sexual Health, Gold Coast Hospital and Health Service, Southport, QLD Australia; 10 Patient Safety and Quality Clinical Nurse Consultant, Gold Coast Hospital and Health Service, Southport, QLD Australia; 11 Nursing and Midwifery Education and Research Unit, Gold Coast Hospital and Health Service, Southport, QLD Australia

**Keywords:** Hospice care, Kennedy terminal ulcer, Palliative care, Pressure ulcer, Skin assessment, Skin failure

## Abstract

**Background:**

Some dying individuals can develop skin injuries at the end-of-life (EOL) due to factors associated with the disease processes, aging or both. These EOL wounds, which include Kennedy terminal ulcers, Trombley-Brennan terminal tissue injuries, Skin Changes at Life’s End and end-stage skin failure, have distinguishing features. Yet, they can appear similar to pressure injuries (PIs), making assessment difficult. Compounding this was the lack of clinical assessment tool for EOL wounds. In 2022, we conducted a modified Delphi panel to develop a new EOL wound assessment tool for use in dying adults and established the face and content validity of the items. The new tool does not differentiate between a PI and EOL wound; rather, it aids clinicians’ assessment of EOL wound characteristics and suggests the development of a multidisciplinary management plan. The next step in the tool development is to determine its reliability. The aim of this study was to test the study protocol and interrater reliability of a new EOL wound assessment tool.

**Methods:**

This feasibility study was conducted in dying hospitalised adult patients admitted to medical and palliative care units at three hospitals across southeast Queensland, Australia. We gathered quantitative data according to the study protocol including participant screening, recruitment, consent, data collection and interrater reliability. Our four research assistants (RAs) and an independent blinded outcome assessor were trained in the study protocol and use of the new EOL wound assessment tool. Using a pragmatic approach, patients with a new reported PI were screened for study eligibility. For recruited participants, clinical data, skin blanching, and a deidentified wound photograph were first collected. Next, the RAs used the new tool to assess the patient and the skin to determine the presence of an EOL wound (Yes/No). An off-site independent blinded outcome assessor accessed the participant research data and, using the new tool, undertook the same assessment as the RA. Frequencies and percentages were computed for the feasibility outcomes. Cohen’s kappa statistic was calculated to determine the interrater reliability agreement.

**Results:**

Over 20 months, 140 patients were screened, with 23 (16.4%) eligible for recruitment, exceeding our ≥ 10% target. Ten (43.5%) participants were recruited, which fell short of our ≥ 50% target, with study refusal and imminent death the reasons for non-recruitment. Among the 10 recruited study participants, 13 wounds were observed on the sacrum, coccyx, and lower extremities. The interrater reliability between the two assessors was moderate (*n* = 8/13; 61.5%), with disagreement on five wounds, all located on the heels and toes.

**Conclusions:**

Assessing for EOL wounds in dying patients is a clinical imperative. With minor study protocol adjustments, such as having two clinicians concurrently undertake independent wound assessment and only recruiting from palliative care units, conducting a larger multisite study testing the inter- and intrarater reliability of the new EOL wound assessment tool is feasible.

**Supplementary Information:**

The online version contains supplementary material available at 10.1186/s12904-025-01853-9.

## Background

The skin is the body’s largest organ and some individuals at the end of their life can develop skin injuries “due to the natural process of disease progression or age (usually in palliative care)” [1] (p. 76) in the hours, days, weeks and months before death [1, 2]. Since 1989, these end-of-life (EOL) wounds have been referred to in the literature as Kennedy terminal ulcer (KTU), 3:30 syndrome [3], Kennedy lesion, Trombley-Brennan terminal tissue injuries (TB-TTI) [4], Skin Changes at Life’s End (SCALE) [2, 5] and end-stage skin failure [6, 7]. The prevalence and incidence of these EOL wounds in any healthcare setting are poorly understood, with estimates ranging from 2.0 to 56.0% [8, 9].

Intense debate and controversy surround these wounds in terms of their aetiology, nomenclature and preventability [1, 5, 10]. This is partly fuelled by the lack of awareness and acknowledgement of these wounds [11] and the paucity of clinical research [1, 12]. The precise aetiology of these wounds is unknown, with multiorgan failure, hypoperfusion, poor nutrition, and low serum albumin hypothesized as playing a role [2, 5, 13, 14]. Sibbald and Ayello’s 2022 survey of 145 wound care professionals found most considered local ischaemia and hypoperfusion contributed to the development of these wounds, with pressure a less likely cause [5]. In 2022, García-Fernández et al. [1] proposed a new conceptual framework for ‘*skin injuries associated with severe-life threatening situations’*, with two sub-types: skin injuries associated with either multiple organ dysfunction syndrome or severe vasoconstriction. The authors propose skin injuries associated with multiple organ dysfunction include KTU, TB-TTI or 3:30 syndrome, which they state are mostly irreversible, defined as unpredictable and unavoidable [1]. Demonstrating progress in the field, in October 2023, the Centers for Medicare and Medicaid Services revised Section M-skin conditions in the *Long Term Care Facility Resident Assessment Instrument (RAI) User’s Manual* [15], recognising these wounds are different from pressure injuries (PIs). Rigorous empirical evidence is needed to improve clinical understanding of these wounds. In the meantime, expert opinion is used to inform practice and policy [16, 17].

Assessing for EOL wounds including KTU [3], TB-TTI [4] and SCALE [2] is complex. This requires a detailed patient medical history including medical diagnosis; confirmation by the healthcare team that the patient is in the terminal phase of their life; ascertaining any precursors to the skin injury; and evidence of implementing appropriate PI prevention practices [7, 16]. EOL wounds have distinct development patterns and wound characteristics. Firstly, these wounds only develop in some dying patients. A distinguishing feature of EOL wounds is their rapid and sudden development. The skin injury can range from non-blanching erythema with intact skin or quickly develop to a deep open ulcer within hours or days [18]. They may appear red, black, or maroon in colour; be pear, horseshoe, or butterfly shaped; and develop on the sacrum, buttocks, spine, and extremities [3, 4]. Furthermore, identifying EOL wounds can be challenging, with evidence suggesting some clinicians, especially novices, lack an awareness of these wounds [11], highlighting the need for targeted education and training [19–21]. In contrast, many experienced clinicians, especially those working in palliative care, have long acknowledged the existence of EOL wounds [5].

Evidence from two systematic reviews revealed that an assessment tool for EOL wounds was non-existent [12, 19], prompting our team to develop one using the limited available evidence and a Delphi panel of experts in the field [22]. Our tool, developed in 2020, is named an ‘End-of-life wound assessment tool’, with the face and content validity of the tool items established by the Delphi panel. The tool intends to aid clinicians’ assessment of the distinct development patterns and characteristics of EOL wounds and suggests developing a multidisciplinary management plan that aligns with patients’ needs and preferences, including possible referral to clinical specialists (e.g., wound, psychological). It is important to note, the new tool does not differentiate between a PI and EOL wound; rather, prior to using the tool, the clinician would have already established the patient’s injury/wound was *NOT* a PI. In the tool, the term EOL wound was defined as a sudden unavoidable skin injury that developed rapidly and includes KTU, TB-TTI and SCALE. The tool, consisting of nine items across three sections: (1) screening, (2) assessment, and (3) confirmation and management. Section 1 involves patient screening using three ‘Yes/No’ questions: (1) the healthcare team assessed the person as dying; (2) the patient has been receiving regular PI prevention strategies as determined by the healthcare team; and (3) did the wound suddenly develop in the previous 24 h. A ‘No’ response to any screening question indicates the tool should not be used. If the clinician responds ‘Yes’ to all three screening questions, they proceed to Sect. 2 (assessment). The assessment section has five wound characteristics (i.e., location, appearance, shape, colour, speed of change) and descriptors, with the clinician responding ‘Yes/No’ to each item. Two or more ‘Yes’ responses in Sect. 2 directs the clinician to Sect. 3. In this section, the clinician confirms if the wound is an EOL wound (‘Yes/No’), and if ‘Yes’, development of a multidisciplinary wound management plan is suggested. The goal of EOL wound care and management should be based on patient preferences, maintaining dignity, and adapting care to their changing needs [20]. This includes providing optimal wound care, monitoring wound healing goals, exudate and odour management, symptom management and pain relief [11, 20].

Acknowledging there is limited EOL wound research, this newly developed new tool contributes to progressing the field by aiding clinicians’ assessment and management of these wounds [22]. With growing research into EOL wounds, we anticipate future modifications to our tool. Testing the interrater reliability of the new tool is necessary to establish its credibility [23, 24]. Nonetheless, we anticipated several potential challenges. First, EOL wounds develop quickly and appear within the hours or days before death, which could make it difficult to recruit study participants. Second, evidence suggests conducting clinical research with dying individuals can result in high participant refusal rates and gatekeeping by clinicians and managers, impacting study recruitment [25]. Therefore, this feasibility study aimed to test the study protocol and interrater reliability of the new EOL wound assessment tool [24]. The study findings will inform the development of a study protocol and sensitive recruitment procedures, data collection, and staff training for a larger future observational study that will determine the tool’s inter- and intrarater reliability in dying hospital patients.

## Methods

Using a pragmatic approach, this feasibility study, conducted between March 2021 and November 2023, gathered study protocol quantitative data in hospitalised dying adult patients with a newly reported PI. Feasibility studies are useful in testing aspects of the methodology, obtaining stakeholder support for a larger study, or determining the ability to collect data on study variables [26]. The feasibility research aims were to:

i. Test participant screening procedures (i.e., study inclusion and exclusion criteria).
ii. Test the recruitment procedure (i.e., how participants were identified, approached, and recruited).
iii. Test the feasibility of collecting wound photographs for interrater reliability testing.
iv. Describe who provided study consent: dying patient, family member, legal guardian.
v. Test the interrater reliability processes for the EOL wound assessment tool.

The study outcomes are outlined in Table 1.

**Table 1:** Study feasibility outcomes

Primary outcomes	Secondary outcomes
1. Eligibility: ≥10% of screened patients with a new reported PI 2. Recruitment: ≥50% of eligible patients are recruited 3. Digital photographs: ≥95% of recruited participants’ PI	1. Number of eligible dying adult patients, family members, or legal guardians who provided study consent. 2. Number of eligible dying adult patients who died/near death before study recruitment. 3. Interrater reliability: ≥90% agreement between raters of wounds

The study reporting followed the Strengthening the Reporting of Observational Studies in Epidemiology (STROBE) guidelines [27]. Ethical approvals were obtained from the relevant hospital and university Human Research Ethics Committees (hospital: HREC/2020/QGC/54403; university: 2020/379).

### Setting

The study settings were acute adult medical and palliative care units at three Australian healthcare facilities (Gold Coast University Hospital, Robina Hospital and Queen Elizabeth II Hospital) located in Queensland’s southeastern region. We recruited seven clinical units (one palliative care unit; six medical units) at Gold Coast University Hospital, five clinical units (one palliative care unit; four medical units) at Robina Hospital, and one palliative care unit at Queen Elizabeth II Hospital. These clinical specialties were selected because of the higher reported PI incidence rates and the larger number of dying patients cared for in these units compared to surgical units. Collectively, these hospitals have about 1,500 beds and provide a range of acute healthcare services (emergency, medical, surgical, palliative, maternity and mental health) to a large and diverse population [28]. Prior to data collection, Chief Investigators [SL, JH, GRB, TH and JS] delivered study information sessions to the nurses in the recruited units.

### Sample and recruitment

As previously stated, the new EOL wound assessment tool was designed to be used only on dying patients suspected of having an EOL wound, including KTU, TB-TTI or SCALE. We recruited the sample from 13 clinical units at three hospitals, which made identifying these potential participants practically impossible. Hence, we identified dying adult patients (aged ≥ 18 years) with a new PI (any stage) reported in the clinical incident management database (RiskMan) in the previous 24 h as eligible to be invited to participate. Participants were excluded if written consent could not be obtained from the patient, family member or legal guardian. The nature of the study and the lack of hypothesis testing means that our sample size calculation used a pragmatic approach based on patient access and study resources [24]. For feasibility studies, a sample size of between 10 and 50 participants is considered sufficient [24]; therefore we aimed to recruit a consecutive sample of 30 dying adult hospital patients meeting the study criteria, or 10 participants per hospital site.

Registered nurses with 1–3 years of clinical experience in palliative care were recruited and trained as Research Assistants (RAs). In addition, a registered nurse who was a palliative wound expert and independent of the research team was also recruited and trained as an off-site independent blinded outcome assessor. A 4-hour training package was delivered by the Chief Investigator [SL] to the RAs and blinded outcome assessor and included the differences between PIs and EOL wounds, the study protocol, data collection including wound photography, and use of the EOL wound assessment tool. After the training, interrater reliability among the RAs, blinded outcome assessor and Chief Investigator [SL] was established by assessing EOL wounds published in the literature [29] to achieve a 0.8 kappa coefficient [30]. If this was not achieved, additional training and wound assessments were performed. Data collection commenced after training and a kappa of 0.8 or greater was achieved.

Using the study criteria, the RAs identified eligible participants in one of two ways. The primary approach involved screening the RiskMan incident reporting database for new PI from the recruited units in the previous 24 h. When potential participants were identified, the RA contacted the nurse unit manager and verbally confirmed that the identified patient was receiving EOL care. In the second approach, the nurse unit manager (or delegate) from the recruited units identified potential patients and directly contacted Chief Investigators [SL] while the clinical staff concurrently entered the RiskMan PI incident data. Obtaining study consent varied depending on the patient’s clinical condition. For conscious patients, the nurse unit manager introduced them to the RA who provided a plain language study overview. Patients were advised of the type of data that would be collected and how it would be used. The RA answered their questions and obtained a written consent from willing participants. For sedated or unconscious patients, the nurse unit manager introduced the RA to the next of kin or legal guardian at the bedside. In a private location, the RA provided them with a plain language study overview, including the type of data collected and how it would be used. All questions were answered and, if willing, a written consent was obtained on behalf of the participant. All participants and legal guardians were reminded of their right to withdraw from the study at any time.

### Data collection

Our new End-of-life wound assessment tool was used to test the interrater reliability processes. In clinical practice, the tool would be used by clinicians if they suspect a dying patient had developed an EOL wound and *NOT* a PI. Our new tool does not differentiate between a PI and EOL wound; rather, it aids clinicians’ assessment of the wound characteristics and suggests the need for developing a multidisciplinary management plan.

Between March 2021 and November 2023, we undertook 20 months of data collection; March–August 2021 (6 months), June–September 2022 (4 months) and February–November 2023 (10 months funded period). During data collection, the RA collected anonymous, deidentified participant data and entered it directly into the Research Electronic Data Capture (REDCap^®^) platform [31] hosted at the university. Baseline data (age, sex, primary diagnosis, number of comorbidities) were gathered from participants’ electronic medical files. Using the EOL wound assessment tool [22], the RA examined the patient file for documented evidence that the healthcare team confirmed the patient was in the EOL phase where death was likely in the following hours, days, weeks or months [32]. They also confirmed from the patient’s medical file documented evidence of the delivery of regular PI prevention strategies, such as repositioning, before reporting the new PI in RiskMan. The RA conducted an independent bedside clinical assessment of the reported PI including a visual inspection of the anatomical location and wound characteristics such as shape, colour, and degree of skin loss, if any. A 3-second skin blanching test adjacent to the wound/injury was completed to assess for reperfusion. Finally, a de-identified colour digital photograph of the new PI was taken. Using the gathered data, the trained RA determined if the new PI was an EOL wound (Yes/No) and documented the outcome of their assessment in REDCap^®^. After data collection, the independent blinded outcome assessor reviewed the data and digital photographs and determined if the wound was an EOL wound (Yes/No). The assessments of the RA and blinded outcome assessor were used to calculate the interrater reliability between the raters.

### Data analysis

Using IBM SPSS Statistics (Version 29.0) [33], the study data were cleaned and tested for accuracy, distribution, and missing values. Frequencies and percentages were computed for the primary and secondary feasibility outcomes. Demographic data were analysed using descriptive statistics. Normally distributed continuous variables were reported using mean and standard deviation (SD), while median and interquartile range (IQR) were used to report nonnormally distributed variables. Categorical variables were reported using frequencies and percentages. Cohen’s kappa statistic was computed to determine the inter-rater reliability agreement with the binary variable: EOL wound (Yes/No) and reported using 95% confidence intervals with a p-value of < 0.05 indicating statistical significance.

## Results

**Fig. 1:**
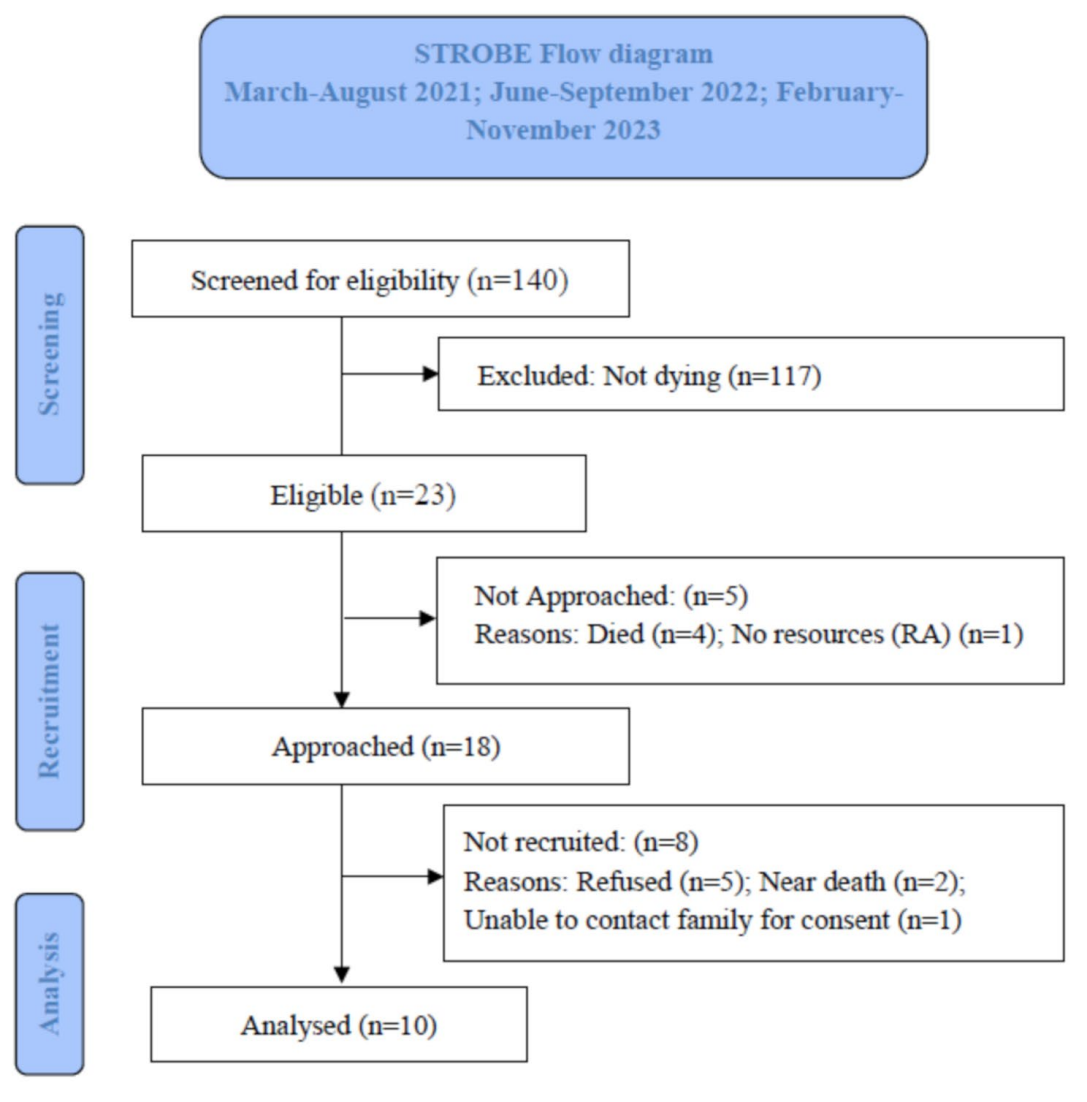
Participants recruitment STROBE flow diagram

Across the 13 recruited clinical units at the three hospitals, 140 adult inpatients with a new PI reported in the RiskMan clinical incident management database were screened, with 23 (16.4%) meeting the study selection criteria (Fig. 1). Of these, four (17.4%) patients died before the RA could commence recruitment. In total, 18 (78.3%) eligible participants, including two others near death at the time, were approached for study recruitment. Eight (34.7%) patients declined to participate due to reasons such as refusing wound photography, not disrupting the patient’s comfort, and wanting to spend time with their loved ones. Therefore, 10 (43.5%) patients (Gold Coast University Hospital: *n* = 0; 0.0%, Robina Hospital: *n* = 3; 30.0%, Queen Elizabeth II Hospital: *n* = 7; 70.0%) who consented were enrolled in the study.

### Feasibility

We met two of our three feasibility criteria (Table 2). We exceeded our eligibility criterion target (*n* = 23/140; 16.4%) and achieved 100.0% for digital photography data collection. We recruited fewer participants than our target (*n* = 10/23; 43.5%), with the actual or imminent death of eligible patients (*n* = 6; 26.1%) being the main reason for non-recruitment.

**Table 2:** Feasibility results

Criteria and target	Result		Target achieved
*Eligibility*: ≥10% of screened patients with a new reported PI are eligible for recruitment	23/140 (16.4%)	Yes	
*Recruitment*: ≥50% of eligible patients are recruited	10/23 (43.5%)	No	
*Digital photography*: ≥95% of recruited participants’ PI were photographed	13/13 (100.0%)	Yes	

The sample of 10 (43.5%) dying adults had 13 new wounds reported in RiskMan. Males (*n* = 7; 70.0%) were mostly recruited, and metastatic cancer was the primary diagnosis for most participants (*n* = 8; 80.0%). The median participant age was 74.0 years (IQR: 63;77, range 44–95 years). All but one participant (*n* = 9; 90.0%) were in specialist palliative care units. Six (60%) patients provided written study consent, with the remaining (*n* = 4; 40.0%) obtained from a partner or adult child. The sample and wound characteristics are presented in Supplementary file 1. Most participants (*n* = 7; 70.0%) presented with one new wound, while three (30.0%) participants each had two new wounds. Digital photographs were collected on the 13 (100.0%) wounds.

### EOL wound assessment tool interrater reliability

We did not meet our interrater reliability target of ≥ 90% agreement. Using the new EOL wound assessment tool, the RA assessed all 13 (100.0%) wounds as EOL wounds. Meanwhile, an independent blinded outcome assessor determined eight (61.5%) of the wounds were EOL wounds, with the remainder assessed as a PI (Supplementary file 1). One (10.0%) participant with two wounds was assessed by the independent outcome assessor as having an EOL wound on one anatomical site and a PI on another site. Disagreement between the raters occurred for five (38.5%) wounds, all located on the heels and toes. The interrater reliability (EOL wound Yes/No) between the RA and blinded outcome assessor using Cohen’s Kappa coefficient could not be calculated because no variation was observed in the RA rating data (Yes = 100.0%), resulting in this being handled as a constant in the SPSS analysis [34]. As such, a percentage agreement was only calculated, with a 61.5% (*n* = 8/13) interrater reliability level of agreement, which according to McHugh [34] is considered moderate (cut-off: 0.60–0.79).

### End-of-life wound characteristics

**Fig. 2:**
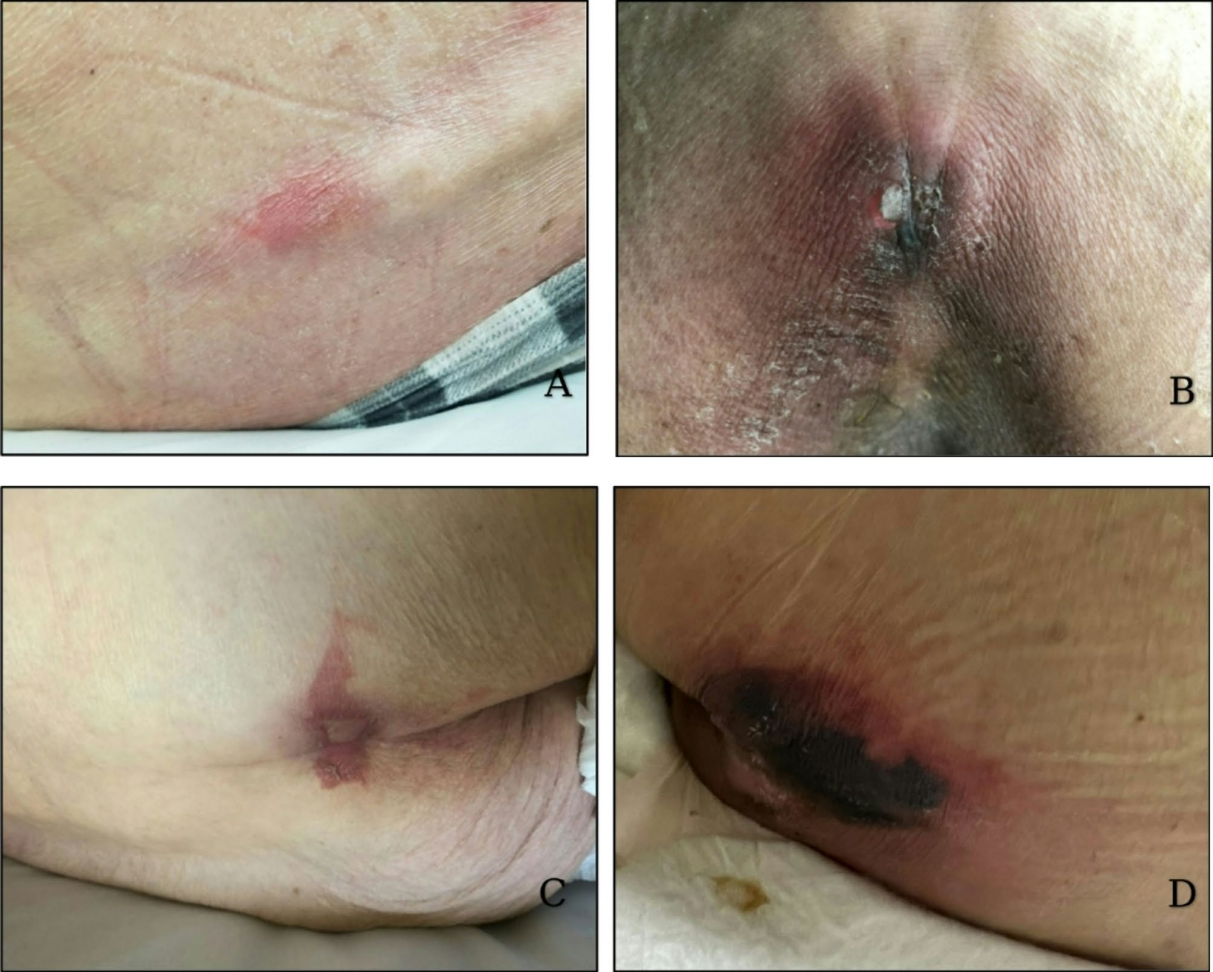
EOL wound photographs. **2A**: Lumbar spine striation; **2B**: Coccyx bilateral butterfly shape; **2C**: Coccyx bilateral butterfly shape with blister; **2D**: Sacro-coccygeal-lumbar horseshoe shape with striations

All of the EOL wounds developed quickly on patient’s sacrum (bilateral) (*n* = 2; 25.0%), lumbar spine (*n* = 2; 25.0%), coccyx (*n* = 1; 12.5%), unilateral buttock (*n* = 1; 12.5%), bilateral buttock (*n* = 1; 12.5%) and upper thigh (*n* = 1; 12.5%). Most (*n* = 7; 87.5%) had a bruise-like appearance, and all (*n* = 8; 100.0%) were nonblanchable. The skin was intact in five (67.5%) of the wounds, and their colour ranged from a single red colour or multiple colours including red, black, pink, purple and maroon, and white in the centre of the wound. Various shapes were observed, with linear striations being the most common (*n* = 5; 62.5%). Other shapes included butterfly (*n* = 2; 25.0%), horseshoe (*n* = 2; 25.0%), and pear (*n* = 1; 12.5%) with and without striations (Fig. 2A-D). We did not collect participant data on wound development and time to death, or indicate if the wounds were KTU, TB-TTI or SCALE because it was outside the study’s scope.

## Discussion

This feasibility study evaluated our study protocol in a sample of dying adult inpatients at three large Queensland hospitals. Although we did not reach our target sample size, we did meet the remaining primary study outcomes. During this study, we gained valuable insights regarding data collection, recruitment and research staff training, which will inform the development of a study protocol for a larger multisite observational study to test the inter- and intrarater reliability of our new EOL wound assessment tool.

Dying adult patients with a new PI were our target population. Using our study criteria, we achieved an eligibility rate of 16.3%, exceeding our ≥ 10% target. We based our study eligibility rate of ≥ 10% on the 2.7% KTU incidence rate reported in cancer patients admitted to hospice care [8], a similar cohort and setting used in our study. Our findings support recent research that found 17.3–19.7% of dying patients developed a KTU [8, 9]. Yet, there is limited reliable prevalence and incidence data on KTU and TB-TTI available in the published literature [35], with estimates varying from 2.0 to 56.0% [12] from a handful of studies across a range of clinical settings [3, 4, 8, 9, 21, 36, 37]. Accurately identifying EOL wounds in dying adults is needed to reduce the misclassification of PI, guide clinical care and resource allocation, and potentially save money for healthcare organisations [12, 20, 35, 38]. As such, further inter- and intrarater reliability testing of our new EOL wound assessment tool is needed. Given our encouraging results, we will retain the study eligibility criteria in a larger research project.

Using an iPad, the RA successfully collected the digital photographs of the reported wounds including those located on the sacrum and buttocks. We attribute this success to our extensive experience of sacral photography in several randomised controlled trials and recruiting registered nurses to gather this data [39–42]. This experience informed the RA training on recruitment, consent and photography including potential ethical and legal issues [43]. During recruitment, the RA and potential participant/family member discussed the role of photography in data collection and analysis, who (i.e., the RA) and how the photographs would be taken (i.e., patient comfort, de-identified, minimal skin exposure, positioning) [44, 45] and its intended use (i.e., manuscripts, conference presentations) [43]. Patient clinical photography is used as standard practice to monitor conditions in dermatology [46], mental health [47], plastic surgery [48] and as a research data collection method [39–42]. Evidence suggests most patients have a positive attitude toward photography for clinical and education purposes [46, 49]; however, for a handful of eligible study patients, privacy concerns regarding the photographs were one reason for recruitment refusal [49]. Clinical experts researching EOL wounds also suggest wound photography can enhance documentation and aid in gaining insights into the pathophysiologic process [5]. We acknowledge the photograph data collection in our study was difficult at times because the RA often gathered this data with minimal assistance while trying to minimise patient discomfort. This meant the quality of some of the collected images could have been affected and is an opportunity for improvement. In a larger study, we will ensure we have the resources to employ two RAs for data collection to improve the wound photography quality and maintain patient comfort.

We did not achieve our recruitment target of ≥ 50% largely because seven eligible patients either died or were close to death at the time of recruitment. Conducting clinical research with dying individuals is challenging [25] and requires the timely identification of living participants and local clinician and manager support [12, 19]. In our study, screening the RiskMan database for potential participants was useful, but we experienced a delay of up to 24 h between the time clinicians entered the data on the new PI to when we conducted the daily screening. The fast-developing nature of EOL wounds in the hours or days prior to a person’s death was a major challenge in our study, which likely contributed to our high rate of ‘near-miss’ recruitments. When conducting research with patients in palliative care units, barriers to recruitment include clinician gatekeeping and ethical concerns about burdening patients can result in high participant refusal rates [25, 50]. Yet, in our study, we received positive support from clinicians and managers in the recruited clinical units, which resulted in some staff directly contacting the researchers about potential participants. All our research team and the RAs were affiliated with the three study sites, either as direct employees or in an adjunct position, and had collegial relationships with many of the staff in the recruited units, which likely contributed to our positive experience. In addition, many of the palliative care clinicians in our recruited units supported our research because they had extensive experience with EOL wounds. In a future study, we will continue to foster collaborations with staff and clinical leaders at potential study sites [50] to build research cross-pollination and develop strategies to increase participant recruitment. In addition, we will invite consumers receiving palliative care and their families as members of our research team and to codesign a future study [51].

We did not achieve our total target sample size of 30 participants. However, our experience of screening 140 patients and achieving a sample of 10 dying patients was considered sufficient to test the feasibility criteria for a larger study. A lack of research funding was the main reason for not achieving our goal, reducing our access to the resources needed to complete the project. This resulted in ad hoc screening, missed recruitment opportunities, and two data collection stoppages lasting 16 months. In 2022, we secured research funding, enabling us to train a team of RAs to collect daily data for 10 months resulting in increased participant identification and recruitment. It is well documented that research into all wounds that develop at the EOL is grossly underfunded, which limits research knowledge development and has clinical implications [19]. Globally, the number of older people and those with chronic health conditions is rapidly growing, placing increased pressure on palliative care in community and healthcare settings [52, 53]. Recruiting dying patients into research projects, as participants or consumers, is often logistically and uniquely challenging [51, 54] and gaining consent directly from study participants is always the preferred legal and ethical option [55]. In our study, 60% of dying patients were willing and able to provide written study consent. This specific population is under-represented in the research literature, so study consent processes require a balance between appropriate protections, and minimising study exclusion, which could limit patient access to safe and effective interventions [55]. Evidence confirms that many dying adults want to participate in clinical research as an act of ‘giving back’ [25, 50]. Consenting dying patients into research projects requires RAs to have specific knowledge and skills on engaging compassionately with potential participants and family members [51, 54]. As such, we recruited registered nurses with palliative care experience and trained them in obtaining consent from dying patients in a tailored and supportive manner, an approach that contributed to our overall study success.

As previously mentioned, our tool does not discriminate between EOL wounds and PIs; rather, it is used if clinicians suspect a wound is an EOL wound, specifically a KTU, TB-TTI or SCALE. EOL wounds are complex and require clinicians to ‘build a picture’ by undertaking a comprehensive patient history, skin and wound assessment. Our moderate (0.61) interrater reliability percentage agreement between the RA and blinded outcome assessor did not reach our target of 0.80. McHugh [34] states that Cohen’s suggested cut offs would have our result falling in the ‘substantial agreement’ band of 0.61–0.80. However, we cautiously interpreted our findings by following McHugh [34] who proposes a Kappa of 0.60–0.79 is considered moderate agreement. We found the difference in ratings occurred at wounds located on the heels and toes, a known location for EOL wound development [12, 19, 35]. Wound assessment is subjective, so differences in clinical judgement and experience between the raters might, in part, explain our findings. The blinded outcome assessor, a palliative care clinical leader, was very familiar with EOL wounds, while our RAs, with 1–3 years of clinical palliative care experience, had limited knowledge of these wounds. The independent outcome assessor determined that one participant with two wounds had an EOL wound as well as a PI. Clinical judgement is a multifaceted and complex concept that requires theoretical knowledge, data, years of clinical experience, and reflection, factors that facilitate pattern recognition in complex medical conditions [56]. Despite extensive training prior to data collection, our less experienced RAs may have lacked the confidence in clinical decision making, which might explain why 100% of their wound assessments affirmed the presence of an EOL wound. Standing’s longitudinal study confirms the role confidence plays in the clinical decision-making skills for first-year registered nurses [57]. In a future larger study, we will obtain resources to support the employment of two experienced palliative care nurses to undertake independent and concurrent bedside clinical assessments, thus replicating real-world clinical practice.

### Limitations

We acknowledge that this feasibility study has limitations. Due to limited funding and resources, our data collection period was extended and interrupted. Also, conducting the research in medical settings did not yield the recruitment results we had hoped for. Future research focussing solely on palliative care units, which have a higher number of dying patients, is one way to increase recruitment and efficiently use research resources. Although our target sample size was not reached, during the 18-month data collection period we garnered sufficient information to rigorously test the study protocol and learn valuable lessons that will inform a larger study. We acknowledge the lack of variation in the outcome data limited our interrater reliability reporting to percentage agreement, which does not take into account if either rater ‘guessed’ the outcome (EOL wound Yes/No) [34]. However, a Cohen Kappa calculation, which accounts for the possibility of guessing, also has limitations by assuming the raters are independent [34]. The outcome assessor was blinded to the RA outcome yet we cannot be certain if the results of both raters were biased based on the study participant [34]. Our efforts to reduce detection bias included independent assessments, blinding of the outcome assessor, and the use of wound photography.

## Conclusions

Assessing for EOL wounds such as KTU, TB-TTI and SCALE is important. This feasibility study tested the study protocols and interrater reliability of our new EOL wound assessment tool in dying hospital patients. Moderate interrater agreement in EOL wounds identification was achieved. Following a few minor protocol modifications, such as having two clinicians concurrently undertaking independent wound assessment and only recruiting from palliative care units, a larger multisite study, testing the inter- and intrarater reliability of the EOL wound assessment tool, is feasible.

## Electronic supplementary material

Below is the link to the electronic supplementary material.

Supplementary Material 1

## Data Availability

The data that support the findings of this study are available on request from the corresponding author, [SL]. The data are not publicly available due to ethical restrictions pertaining to the pri-vacy of research participants.

## Abbreviations

EOL: End-of-life
F: Female
IQR: Interquartile range
M: Male
PI: Pressure injuries
RA: Research assistant
SD: Standard deviation
STROBE: Strengthening the Reporting of Observational Studies in Epidemiology
