# Supplementary material for: Testing the study protocol and interrater reliability of a new end-of-life wound assessment tool: a feasibility study
Source: BMC Palliat Care. 2025 Jul 29;24:216. doi: 10.1186/s12904-025-01853-9 (PMC12309095; doi:10.1186/s12904-025-01853-9)
Supplement: Supplementary file 1 — Supplementary Material 1 [file 12904_2025_1853_MOESM1_ESM.docx]

| **Supplementary file 1:** Sample and wound characteristics (*n* = 10) | | | | | | | | | | | | | |
| --- | --- | --- | --- | --- | --- | --- | --- | --- | --- | --- | --- | --- | --- |
| Age (years) | Sex  (M/F) | | Study consent obtained from | Primary diagnosis | Number of observed wound/s | Clinical staff confirm patient is dying (Yes/No) | Wound developed quickly in the absence of pressure (Yes/No) | Received regular PI prevention care  (Yes/No) | Wound location | Observed wound characteristics | EOL wound or Pressure injury rating | | Inter-rater agreement  (Yes/No) |
|  |  |  |  |  |  |  |  |  |  |  | Research Assistant | Outcome Assessor |  |
| 44 | M | | Patient | Metastatic oesophageal cancer | 1 | Yes | Yes | Yes | Coccyx | - Non-blanchable - Colour: pink, purple and maroon - Shape: butterfly and pear - Wound appearance: stage II PI (full thickness skin loss) | EOL wound | EOL wound | Yes |
| 65 | F | | Patient | Pyloric adenocarcinoma | 1 | Yes | Yes | Yes | Sacro-coccygeal-lumbar region (bilateral) | - Non-blanchable - Colour: red, black, purple, and maroon - Shape: horseshoe and striations - Wound appearance: bruise like appearance (skin intact) and a stage II PI (full thickness skin loss) | EOL wound | EOL wound | Yes |
| 74 | F | | Patient | Metastatic lung cancer | 1 | Yes | Yes | Yes | Sacrum (bilateral) | - Non-blanchable - Colour: pink, purple and maroon - Shape: butterfly - Wound appearance: bruise like appearance (skin intact) and a stage II PI (blister with partial-thickness skin loss) | EOL wound | EOL wound | Yes |
| 77 | M | | Patient | Metastatic prostate cancer | 1 |  | Yes | Yes | Buttock (bilateral) | - Non-blanchable - Colour: red and pink - Shape: three linear striations - Wound appearance: bruise like appearance (skin intact) | EOL wound | EOL wound | Yes |
| 79 | M | | Patient | Bladder cancer | 1 | Yes | Yes | Yes | Lumbar spine | - Non-blanchable - Colour: red - Shape: linear striation - Wound appearance: bruise like appearance (skin intact) | EOL wound | EOL wound | Yes |
| 95 | F | | Adult child | Sudden clinical deterioration | 2 | Yes | Yes | Yes | Right buttock (unilateral) | - Non-blanchable - Colour: red - Shape: one linear striation - Wound appearance: bruise like appearance (skin intact) | EOL wound | EOL wound | Yes |
|  |  |  |  |  |  |  |  |  | Right posterior upper thigh | - Non-blanchable - Colour: red - Shape: two linear striations - Wound appearance: bruise like appearance (skin intact) | EOL wound | EOL wound | Yes |
| 71 | M | | Partner | Metastatic pancreatic cancer | 2 | Yes | Yes | Yes | Sacrum (bilateral) | - Non-blanchable - Colour: red, deep darkening skin with white center - Shape: horseshoe - Wound appearance: bruise like appearance (skin intact) | EOL wound | EOL wound | Yes |
|  |  |  |  |  |  |  | Yes | Yes | Lateral right foot and heel | - Non-blanchable - Colour: pink, purple and maroon - Shape: linear striations - Wound appearance: bruise like appearance (skin intact) | EOL wound | Pressure injury | No |
| 59 | M | | Partner | Metastatic cancer | 1 | Yes | Yes | Yes | Left greater metatarsal joint | - Non-blanchable - Colour: red, pink, purple and maroon - Shape: circular - Wound appearance: stage II PI (blister with partial-thickness skin loss) | EOL wound | Pressure injury | No |
| 74 | F | | Patient | Metastatic Colorectal Cancer | 2 | Yes | Yes | Yes | Left heel | - Non-blanchable - Colour: pink, purple and maroon - Shape: round - Wound appearance: bruise like appearance (skin intact) | EOL wound | Pressure injury | No |
|  |  |  |  |  |  |  | Yes | Yes | Right heel | - Non-blanchable - Colour: pink, purple and maroon with a white center - Shape: pear - Wound appearance: stage II PI (full thickness skin loss) | EOL wound | Pressure injury | No |
| 77 | M | | Partner | Sepsis | 1 | Yes | Yes | Yes | Right heel | - Non-blanchable - Colour: pink, purple and maroon - Shape: circular - Wound appearance: bruise like appearance (skin intact) | EOL wound | Pressure injury | No |
|  | | Abbreviations: EOL: end-of-life; F: female; M: male; PI: pressure injury | | | | | | | | | | | |
